# Temperature and soil moisture manipulation yields evidence of drought‐induced pollen limitation in bee‐pollinated squash

**DOI:** 10.1002/ece3.11400

**Published:** 2024-06-02

**Authors:** Jess Gambel, David A. Holway

**Affiliations:** ^1^ Division of Biological Sciences University of California at San Diego La Jolla California USA; ^2^ Present address: USDA‐Agricultural Research Service Research Participation Program Oak Ridge Institute for Science and Education, Dairy Forage Research Center Madison Wisconsin USA

**Keywords:** climate change, drought, pollen limitation, pollination, squash, warming

## Abstract

Climate change alters environmental conditions in ways that directly and indirectly affect plants. Flowering plants, for example, modify reproductive allocation in response to heat and drought stress, and such changes can in turn affect pollinator visitation and, ultimately, plant reproduction. Although the individual effects of warming and drought on plant reproductive allocation are well known, these factors may interact to influence reproduction. Here, we conducted a fully crossed temperature by irrigation manipulation in squash (*Cucurbita pepo*) to test how temperature and soil moisture variation affect pollinator‐mediated reproduction. To tease apart the direct and indirect effects of temperature and soil moisture, we compared hand‐pollinated plants to bee‐pollinated plants and restricted bee foraging (i.e., pollen transfer) to one experimental group per day. Temperature and soil‐moisture limitation acted independently of one another: warming decreased flower size and increased pollen production, whereas the effects of soil‐moisture limitation were uniformly inhibitory. While treatments did not change squash bee (*Xenoglossa* spp.) behavior, floral visitation by the honey bee (*Apis mellifera*) increased with temperature in male flowers and decreased with soil moisture in female flowers. Pollen deposition by bees was independent of plant soil moisture, yet reducing soil moisture increased pollen limitation. This result stemmed at least in part from the effects of soil‐moisture limitation on pollen viability; seed set declined with increasing deposition of fluorescent pigment (a proxy for pollen) from plants experiencing decreased soil moisture. These findings suggest that the transfer of lower‐quality pollen from plants experiencing soil‐moisture limitation led to drought‐induced pollen limitation. Similar effects may occur in a wide variety of flowering plant species as climate warming and drought increasingly impact animal‐pollinated systems.

## Graphical abstract

Climate change alters environmental conditions in ways that directly and indirectly affect plants. We investigated how warming and drought impacted reproduction in bee‐pollinated squash using a fully crossed factorial temperature by irrigation manipulation experiment. By teasing apart the direct (in hand‐pollinated plants) and indirect (in bee‐pollinated plants) effects on plant reproductive success, we found that pollinators likely mediated drought‐induced pollen limitation by transporting and depositing pollen from plants experiencing soil‐moisture limitation.

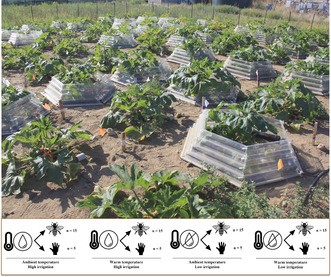

## 1 INTRODUCTION

Over the last century, anthropogenic climate change has raised global surface temperatures more than 1°C, resulting in more frequent weather extremes (Lee et al., 2023), including severe droughts (Pachauri et al., 2014; Slette et al., 2019). Hot and arid conditions affect organisms in a variety of ways (Lee et al., 2023) with flowering plants often responding through changes in floral traits. Heat stress, for example, can diminish flower size (de Manincor et al., 2023; Hoover et al., 2012) yet have opposing effects on flower number (de Manincor et al., 2023; Descamps, Jambrek, et al., 2021; Hoover et al., 2012; Moss & Evans, 2022). Warming may also reduce pollen viability (Koti et al., 2005), increase nectar concentration (de Manincor et al., 2023), and generate variable effects on nectar production (de Manincor et al., 2023; Descamps, Jambrek, et al., 2021; Hoover et al., 2012; Moss & Evans, 2022). Soil‐moisture limitation, in contrast, mostly reduces reproductive allocation (Descamps, Quinet, & Jacquemart, 2021; Höfer et al., 2022; Wu et al., 2022). To date, few experimental studies consider the joint effects of warming and soil‐moisture limitation, but variation in both of these conditions can decrease floral abundance (Schuchardt et al., 2021), flower size (Descamps et al., 2018, 2020), pollen viability (Descamps et al., 2018), nectar production (Descamps et al., 2020), and sugar content (Descamps et al., 2018) in particular plant species. Given that warming and soil‐moisture limitation can covary and give rise to interactive impacts on plant reproduction, considering the joint effects of these two stressors together seems warranted.

By modifying floral provisioning, warming and soil‐moisture limitation may impact rates of pollinator visitation in flowering plants. Heat stress in plants can lower the frequency (de Manincor et al., 2023; Descamps, Jambrek, et al., 2021) and duration (de Manincor et al., 2023) of bee visits and lead to complicated effects on plant–pollinator networks (Moss & Evans, 2022). Similarly, plants subjected to drought conditions can also experience reduced bee visitation (Al‐Ghzawi et al., 2009; Burkle & Runyon, 2016; Gambel & Holway, 2023; Glenny et al., 2018; Rering et al., 2020). In some cases, however, pollinators may visit flowers more often but for shorter lengths of time because of reductions in floral resources caused by soil‐moisture limitation (Waser & Price, 2016). In other instances, bee preferences for particular floral characteristics may supersede effects of decreased soil moisture (Gallagher & Campbell, 2017). Heat and aridity in combination can reduce bee foraging in flowering plants (Descamps et al., 2018), but few studies have examined the joint effects of warming and soil‐moisture limitation on pollinator visitation. Furthermore, changes in pollinator behavior resulting from plants experiencing physiological stress could lead to pollen limitation (i.e., insufficient pollen receipt by the stigma of a female flower), either through the transport of less pollen or pollen of reduced viability (Al‐Ghzawi et al., 2009; Rering et al., 2020; Wu et al., 2022).

Recent attention to how climate change influences plant–pollinator interactions recognizes that warming or drought can disrupt interactions among plants and pollinators with potentially negative consequences for plant reproduction (de Manincor et al., 2023; Gambel & Holway, 2023; Moss & Evans, 2022; Rering et al., 2020). To date, research has mostly addressed how variation in temperature (de Manincor et al., 2023; Moss & Evans, 2022) or soil moisture (Al‐Ghzawi et al., 2009; Rering et al., 2020; Waser & Price, 2016) impact plant reproductive allocation and how pollinator behavior responds to changes in floral resources. Direct effects (i.e., plant responses) and indirect effects (i.e., pollinator responses) can interact with one another, however, in ways that may ultimately compromise plant reproduction (Gambel & Holway, 2023). Reductions in pollen quantity (number of grains) or quality (viability) caused by warming or soil‐moisture limitation, for example, could result in pollen limitation even in cases when rates of pollinator visitation remain unchanged. To separate the direct effects of environmental stress on reproduction from those effects resulting from pollinator visitation, it is necessary to compare reproductive performance between plants pollinated by insects and plants pollinated by hand (pollinators excluded). Teasing apart these direct and indirect effects will clarify if plants could become more pollen‐limited in hotter, drier climates.

In the present study we focus on the effects of experimental warming and soil‐moisture limitation on reproduction in the squash *Cucurbita pepo* (Cucurbitaceae). We conducted a factorial temperature by irrigation manipulation of squash plants under field conditions (i) to assess the direct effects of environmental variation on plant reproduction, and (ii) to examine how reproduction was affected by pollinator responses to floral traits that might be compromised by altered physical conditions. Importantly, we designed our experiment such that only plants experiencing the same combinations of temperature and irrigation were accessible to pollinators on a given day (i.e., plants only received pollen from plants in the same experimental group). We tested four predictions. (i) Increased temperatures and decreased soil moisture reduce plant reproductive allocation through changes to floral traits. To test this prediction, we examined how flower size, flower number, nectar production, and pollen production responded to experimental warming and soil‐moisture limitation. (ii) Changes in floral resources caused by warming and soil‐moisture limitation, in turn, affect pollinator visitation and behavior. To test this second prediction, we determined how pollinators responded to plants that experienced different levels of warming and soil‐moisture limitation. (iii) Pollinators impact how plants respond to stress via effects on stigmatic pollen deposition. To test this third prediction, we measured pollen limitation as a function of physical conditions. (iv) If warming and soil‐moisture limitation influence pollen limitation, then pollen deposited by pollinators would be expected to vary in quantity or quality as a function of temperature and irrigation level. We tested this final prediction using results from prediction (i) and data from a second experiment that specifically tested how variation in physical conditions affected pollen viability. This study is one of few (Gambel & Holway, 2023) that attempts to separate the direct effects of warming and soil‐moisture limitation on plant reproduction from those effects that result from pollinator foraging behavior. Given that warming and soil‐moisture limitation commonly impact reproductive allocation in animal‐pollinated plant species, pollinator‐mediated effects are likely widespread.

## 2 METHODS

### 2.1 Study system

We grew *Cucurbita pepo* plants (Honey Bear F1 acorn squash, Johnny's Selected Seeds©) at the University of California, San Diego Biology Field Station (32.885778° N, 117.229778° W) in individual 1.2 m × 1.2 m plots during the summers of 2016 and 2018. *Cucurbita* produce separate male (staminate) and female (pistillate) flowers with large, colorful corollas and ample, sugar‐rich nectar (Hurd et al., 1971). Individual flowers remain open and receptive to pollinators for only one morning; flowers can thus be collected and measured the same day (Tepedino, 1981). Owing to the production of separate male and female flowers, all *Cucurbita* species require pollinators to set seed (Artz & Nault, 2011; Hoehn et al., 2008; Hurd et al., 1971). Like other *Cucurbita*, *C. pepo* attracts a variety of insect pollinators, primarily bees, which include generalists, such as the western honey bee (*Apis mellifera*), and specialists, such as squash bees (*Xenoglossa* [formerly *Peponapis* and *Xenoglossa*]; Freitas et al., 2023), that require pollen from cucurbits to reproduce (Hurd et al., 1974). For the above reasons and because of the economic importance of cultivated squash (Gallai et al., 2009; McGrady et al., 2020), interactions between *Cucurbita* and their pollinators are extensively studied (Artz & Nault, 2011; Delgado‐Carrillo et al., 2018; Hoehn et al., 2008; Hurd et al., 1971; Hurd et al., 1974; Tepedino, 1981), including in the context of how climate change affects squash reproduction (Gambel & Holway, 2023; Hoover et al., 2012).

### 2.2 Experiment I: Effects of warming × soil‐moisture limitation on bee visitation and plant reproduction

From June through September 2016, we conducted a fully crossed, two‐way factorial temperature (warmed, ambient) by irrigation (high, low) experiment with 20 *C. pepo* plants in each of four experimental groups (see Appendix S1). Individual plants in each group were spatially interspersed within the field. To elevate the ambient air temperature around squash plants, we used passive, open‐top warming chambers (Godfree et al., 2011) that fit over individual plants (see Appendix S1). Thermochron iButtons© that recorded hourly temperature readings (Godfree et al., 2011) for each plant throughout the season indicated that mean daily temperatures were 2.5°C higher for plants in the warmed treatment group compared to plants in the ambient treatment group (one‐way ANOVA: *F*_1,78_ = 500.80, *p* < .0001; ambient: 23.36 ± 0.072 [mean ± SE]°C, warmed: 25.88 ± 0.086°C; see Appendix S1). This temperature increase falls within the projected rise of the global mean surface temperature by the end of the current century (Lee et al., 2023).

We used drip irrigation to manipulate soil moisture levels experienced by plants (as in Gambel & Holway, 2023; see Appendix S1). The irrigation treatment included either 2.0 L water/plant/day for the high‐irrigation group or 0.67 L water/plant/day for the low‐irrigation group. For plants in the high‐irrigation group, water did not appear to limit growth, whereas floral resources were compromised for plants in the low‐irrigation group (Table 1); low levels of irrigation thus induced soil‐moisture limitation (Slette et al., 2019). We used a FieldScout TDR 100© soil moisture meter to determine each plant's mean volumetric water content (VMC%) during the season (as in Waser & Price, 2016): mean VMC was 10% lower for plants in the low‐irrigation group compared to plants in the high‐irrigation group (two‐way ANOVA: *F*_1,76_ = 93.92, *p* < .0001; low: 36.02 ± 0.72 [mean ± SE] VMC%, high: 46.32 ± 0.77 VMC%; see Appendix S1). Soil moisture levels, however, were highly variable within each irrigation group; thus, we treat plant soil moisture as a continuous variable in all statistical analyses (as in Gallagher & Campbell, 2017) but retain irrigation group designations (i.e., high, low) in the organization of the experiment. For the observed, plant‐level measures of soil moisture, the factors of temperature and irrigation level were independent of one another (see Appendix S1). All analyses are restricted to data collected from the month of July (during the height of flowering and pollinator visitation; see Appendix S1).

**TABLE 1:** The effects of soil moisture, temperature, and their interaction on floral traits in bee‐pollinated *Cucurbita pepo* (*n* = 60 plants) in *Experiment I*.

Response Variable	Factor	*df*	*F*	*p*	$R_{\mathrm{adj}}^{2}$
Total male flowers	Mean soil moisture	1,56	0.30	.59	.039
	Temperature	1,56	2.17	.15	
	Soil moisture × temperature	1,56	2.94	.092	
Total female flowers*	Mean soil moisture	1,56	0.0030	.96	.00
	Temperature	1,56	0.020	.89	
	Soil moisture × temperature	1,56	0.43	.52	
Male flower size (corolla width in mm)	**Mean soil moisture**	**1,56**	**22.72**	**<.0001**	**.32**
	**Temperature**	**1,56**	**5.72**	**.020**	
	Soil moisture × temperature	1,56	2.22	.14	
Female flower size (corolla width in mm)	**Mean soil moisture**	**1,55**	**21.11**	**<.0001**	**.33**
	**Temperature**	**1,55**	**10.12**	**.0024**	
	Soil moisture × temperature	1,55	0.029	.87	
Nectar volume (μL) in male flowers	**Mean soil moisture**	**1,56**	**6.11**	**.017**	**.13**
	**Temperature**	**1,56**	**4.09**	**.048**	
	Soil moisture × temperature	1,56	1.65	.20	
Nectar volume (μL) in female flowers	Mean soil moisture	1,55	3.25	.077	.078
	Temperature	1,55	3.11	.084	
	Soil moisture × temperature	1,55	1.58	.21	
Nectar concentration (BRIX) in male flowers	Mean soil moisture	1,56	2.04	.16	.14
	Temperature	1,56	0.46	.50	
	**Soil moisture × temperature**	**1,56**	**10.28**	**.0022**	
Nectar concentration (BRIX) in female flowers^&^	Mean soil moisture	1,55	0.0040	.95	.00
	Temperature	1,55	2.91	.094	
	Soil moisture × temperature	1,55	0.038	.85	
Pollen weight (mg) in male flowers^+^	Mean soil moisture	1,56	0.10	.75	.045
	**Temperature**	**1,56**	**5.09**	**.028**	
	Soil moisture × temperature	1,56	0.60	.44	

*Note*: Models are ANCOVAs. To improve normality of the residuals, traits that were right‐skewed were either log_10_ (*) or square‐root (^+^) transformed, while traits that were left‐skewed were square transformed (^&^). All plant measurements (except flower count) were averaged across the flowering season. Results in bold are statistically significant at *α* = .05. Abbreviations: *df*, degrees of freedom; $R_{\mathrm{adj}}^{2}$, adjusted *R* ^2^ value.

To determine how temperature and irrigation affected reproductive allocation, we measured floral traits from a subset of female and male flowers from which pollinators were excluded using Seedburo Treated S27 Shoot Pollinating Bags (5 × 2.5 × 18 cm; Stoner & Eitzer, 2012; see Appendix S1). For these unvisited flowers, we measured flower size (male flowers, *n* = 79 plants; female flowers, *n* = 75 plants), nectar volume (male flowers, *n* = 79 plants; female flowers, *n* = 75 plants), nectar concentration (male flowers, *n* = 78 plants; female flowers, *n* = 75 plants), and pollen weight (male flowers, *n* = 79 plants).

To assess how the temperature and irrigation treatments affected pollinator behavior, we used video cameras to record bee visitation to flowers. We first assigned plants to either a bee‐pollinated group (*n* = 15 plants) or a hand‐pollinated group (*n* = 5 plants) within each experimental treatment (see Appendix S1). Individual plants in these two groups were spatially interspersed within the field. Bee‐pollinated plants were open to pollinators subject to the constraint that only plants within the same experimental group (e.g., warmed + low irrigation) were accessible on a given day; all other open flowers were bagged. This approach ensured that bee‐pollinated plants only received pollen from plants in the same experimental group. For plants in the warmed treatment, we temporarily removed open‐top chambers from plants as needed to allow free access by pollinators. We randomly selected bee‐pollinated plants in each experimental group and, each morning of the experiment, video‐recorded a subset of open male flowers (*n* = 33 plants) and open female flowers (*n* = 27 plants) for 1.5 h (see Appendix S1). In each video of a flower, we determined visitation rate (visits/min/flower), and for each visit measured the time that the focal bee spent (i) in contact with the stigma (female flowers) or anthers (male flowers), and (ii) collecting pollen (male flowers). To assess how temperature and irrigation directly affected plant reproduction, we included hand‐pollinated plants by excluding bees from visiting flowers on these plants (as described above; Stoner & Eitzer, 2012). Consequently, flowers on hand‐pollinated plants were only pollinated with pollen from other hand‐pollinated plants within the same experimental group (see Appendix S1).

To ascertain the indirect effects of temperature and irrigation on plant reproduction via pollinator behavior, we compared pollen deposition, fruit set, and seed set between hand‐pollinated plants and bee‐pollinated plants. After squash flowers closed (i.e., early afternoon on the day they opened), we randomly selected pollinated flowers to quantify either (i) pollen deposition, or (ii) fruit and seed set. We estimated pollen deposition as follows. We collected stigmas, stored them in ethanol (Winsor et al., 2000), and stained them with basic fuchsin dye (Kearns & Inouye, 1993). We then calculated the mean (as in Artz & Nault, 2011) number of pollen grains deposited on two out of six stigmatic lobes and added this number to the number of stained grains in solution. All pollen grain counts were made at 50× magnification under a dissecting microscope. Flowers that were not sacrificed to estimate pollen deposition were used to determine fruit and seed set. All plants were given at least one chance to set fruit from a pollinated female flower. When fruit set did occur, we harvested fruit 50 days after pollination (Loy, 2004); this time period was sufficient for seed maturation. After harvested, seeds were dried and weighed. We report total seed weight per fruit per plant as a measure of seed set. Total seed weight per fruit was positively correlated with total seed number per fruit for all plants (Pearson correlation: *r*_77_ = 0.95, *p* < .0001). For plants that set more than one fruit (*n* = 23), we used mean values to estimate total seed weight.

To determine if bee‐pollinated plants experienced pollen limitation, we estimated pollen limitation as L = 1 – (B/H), where B and H represent total seed weight for bee‐pollinated plants and hand‐pollinated plants, respectively (Larson & Barrett, 2000). Here pollen limitation increases as the value of L approaches 1. We estimated L by using total seed weight measurements for each hand‐pollinated plant (i.e., H; *n* = 18) matched to a bee‐pollinated plant (i.e., B; *n* = 18) from the same experimental group (e.g., warmed + low irrigation) and grown under a similar moisture level. For hand (*n* = 5) and bee (*n* = 3) plants with more than one fruit, we used the mean total seed weight value for each plant. As an additional method of estimating pollen limitation, we determined L for all bee‐pollinated plants (*n* = 55) from the total seed weight value for each bee‐pollinated plant (i.e., B) and the mean total seed weight value from the matched hand‐pollinated treatment group (i.e., H).

### 2.3 Experiment II: Effects of soil‐moisture limitation on pollen viability

Based on the results of *Experiment I* (see Results), as well as the findings from previous studies (Al‐Ghzawi et al., 2009; Rering et al., 2020; Waser & Price, 2016), we conducted a second experiment that tested how variation in plant soil moisture affected pollen viability (as in Waser & Price, 2016). *Experiment II* thus did not include a temperature treatment. Moreover, in *Experiment II*, unlike in *Experiment I*, bees moved freely among all plants and could collect and deposit pollen on plants grown under any level of soil moisture. From June through September 2018, we reared squash in an identical manner to that described under *Experiment I*. The irrigation treatment included two levels: 2.2 L water/plant/day (high, *n* = 31 plants) and 0.35 L water/plant/day (low, *n* = 30 plants). Mean volumetric water content for plants in the low‐irrigation group (range: 24%–48%) was 10% less than that of plants in the high‐irrigation group (range: 38%–51%; one‐way ANOVA: *F*_1,89_ = 143.30, *p* < .0001; low: 34.05 ± 0.65 [mean ± SE] VMC%, high: 43.86 ± 0.49 VMC%). All analyses are restricted to data collected from July 21–August 21 during the height of flowering and pollinator visitation (see Appendix S1).

To examine how soil‐moisture limitation influenced pollen viability, we assessed how pollen source (i.e., irrigation group) affected plant reproduction. We used florescent powdered pigments applied to male flowers to both track pollen transfer by bees (Ordway et al., 1987) to the stigmas of female flowers and to serve as a proxy for the number of pollen grains transported (see Waser, 1988) from low‐irrigation and high‐irrigation plants to plants grown along a soil moisture gradient. For this experiment, we collected all pollinated stigmas after first allowing pollen to germinate for 24 h to permit pollen grains to fertilize ovules (*pers. obs*.). Stored stigmas were first counted for the number of deposited pigment particles on entire stigmas using a dissecting microscope at 40x magnification (Waser, 1988). Stigmas were then stained (with basic fuchsin dye; Kearns & Inouye, 1993) and counted at 50x magnification for the number of deposited pollen grains on entire stigmas (Artz & Nault, 2011), including stained grains present in solution. Mean pigment particle count (per stigma per plant) was positively correlated with mean pollen grain count (per stigma per plant) (Pearson correlation: *r*_66_ = 0.83, *p* < .0001). We harvested fruit and measured seed set as in *Experiment I*. Again, we treated plant as the experimental unit of analysis. Five plants did not have the chance to set fruit from a pollinated female flower. For plants that set more than one fruit (*n* = 3), we used mean values for total seed weight (per fruit per plant).

### 2.4 Statistical analyses

We used R version 3.6.1 (R Core Team, 2019) for all data analyses (Gambel, 2024). Our analyses employed *nortest* (Gross & Ligges, 2015) to run tests for normality, *plotrix* (Lemon et al., 2021) to find the standard error of the mean, *ggplot2* (Wickham, 2009) to prepare figures, and *wesanderson* (Ram et al., 2018) to color figures. To test the assumptions of general linear models, we used Bartlett tests to assess homogeneity of variances, inspected q‐q plots to assess normality of errors, and used Shapiro–Wilk tests to check residuals. These tests of assumptions in some cases motivated data transformations (see Results). Raw data are plotted in all figures.

For *Experiment I* we used general linear models to test how soil moisture (mean volumetric water content) and temperature (warmed, ambient) affected total flower number, flower size, nectar volume, nectar concentration, pollen weight, bee visitation and behavior rates in flowers, and pollen limitation. We also used general linear models with soil moisture, temperature, and pollination (bee, hand) as fixed factors to test whether or not treatments affected pollen deposition (general linear model), fruit set (general linear model, family = binomial), and seed set (general linear model). For *Experiment II*, we used a general linear model to assess how the proportion of deposited pigment from plants grown under low irrigation (i.e., pollen source) affected seed set. We ran additional general linear models to determine how stigmatic pollen deposition and plant soil moisture affected seed set (see Appendix S1).

## 3 RESULTS

### 3.1 Experiment I: Effects of warming × soil‐moisture limitation on bee visitation and plant reproduction

**FIGURE 1:**
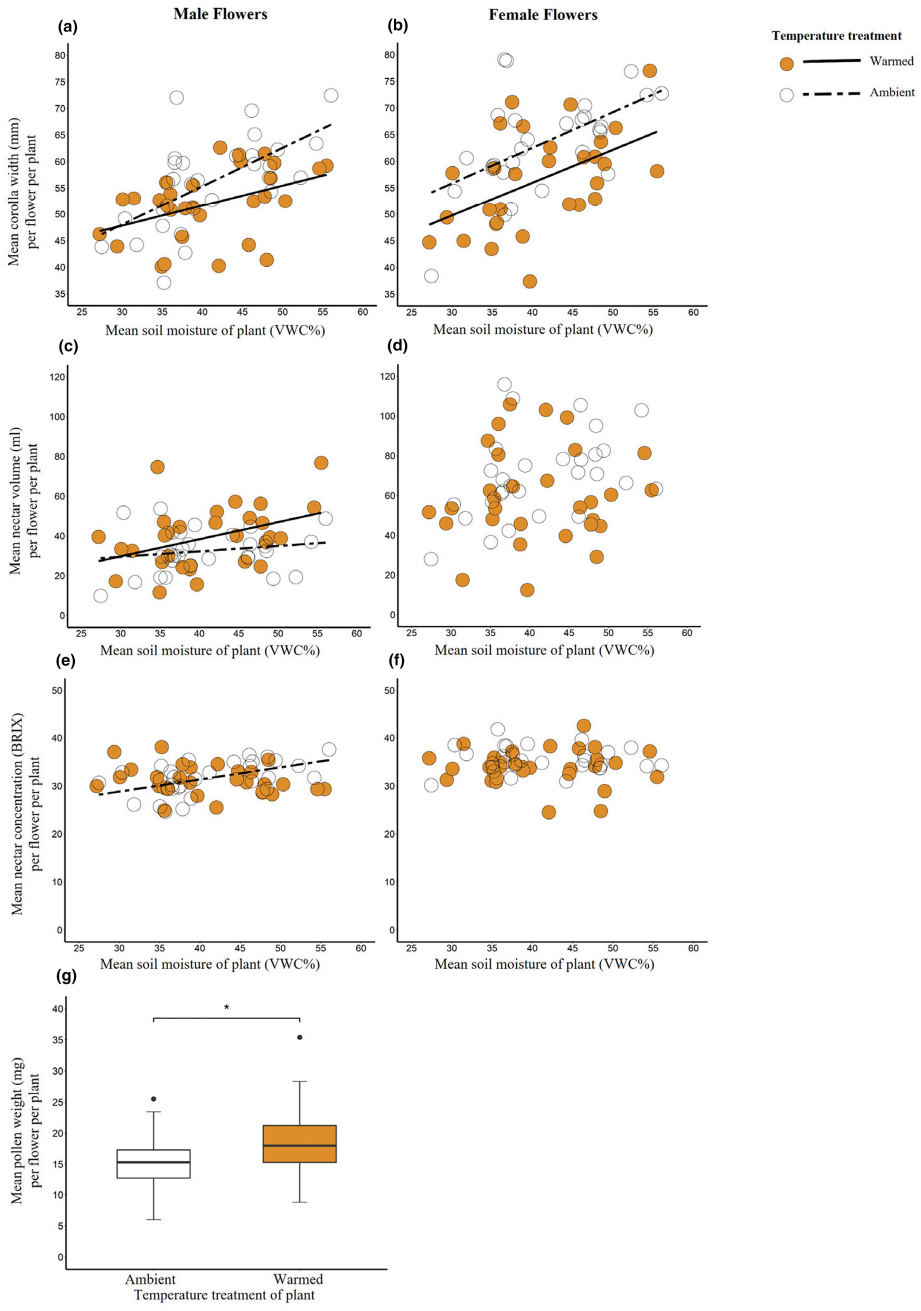
Effects of temperature and soil moisture on flower size (a, b), nectar production (c, d), nectar concentration (e, f), and pollen production (g) for male (a, c, e, g) and female (b, d, f) flowers on bee‐pollinated *Cucurbita pepo* from *Experiment I*. Regression lines in (a)–(f) and asterisk (*) in (g) represent significant relationships; see Results and Table 1 for additional statistical information. For (g), box plots include the interquartile range with median line and whiskers that show quartiles ±1.5× interquartile range; points represent extreme values.

Temperature and soil moisture each affected reproductive allocation in bee‐pollinated squash plants, but these factors generally had independent effects on floral traits (Table 1; see Appendix S1). When effects on flowers were evident, low soil moisture levels, but not warm temperatures, were always inhibitory. Although flower production (per plant) was unaffected by variation in temperature and soil moisture (Table 1; see Appendix S1), flower size decreased with decreasing soil moisture and under warmed conditions (for male flowers, ambient: 56.14 ± 1.56 [mean ± SE] mm corolla width/flower/plant, warmed: 51.97 ± 1.22 mm corolla width/flower/plant; for female flowers, ambient: 63.34 ± 1.68 mm corolla width/flower/plant, warmed: 56.49 ± 1.70 mm corolla width/flower/plant; Table 1; Figure 1a,b; see Appendix S1). For male flowers, nectar volume increased under warmed conditions (ambient: 32.55 ± 1.96 μL/flower/plant; warmed: 39.08 ± 2.86 μL/flower/plant) but decreased with decreasing soil moisture (Table 1; Figure 1c; see Appendix S1). Nectar concentration also decreased with decreasing soil moisture under ambient temperatures in male flowers (simple linear regression: *F*_1,28_ = 10.82, *R* ^2^_adj_ = .25, *p* < .01; Table 1; Figure 1e; see Appendix S1). For female flowers, neither temperature nor soil moisture affected nectar volume (Table 1; Figure 1d; see Appendix S1) or concentration (Table 1; Figure 1f; see Appendix S1). Lastly, warming stimulated pollen production by male flowers (ambient: 15.36 ± 0.83 mg/flower/plant; warmed: 18.45 ± 1.05 mg/flower/plant; Table 1; Figure 1g; see Appendix S1).

Honey bees (*Apis mellifera*) and squash bees (*Xenoglossa* spp.) together accounted for 97% of floral visits by pollinators to squash flowers. The only effects of treatment on visitation rate or pollinator behavior were exhibited by honey bees (see Appendix S1). The rate of *Apis* visitation to female flowers increased with plant soil moisture (*F*_1,23_ = 4.79, $R_{\mathrm{adj}}^{2}$ = .13, *p* < .05; see Appendix S1) and the cumulative time that *Apis* spent collecting pollen in male flowers was higher for warmed plants (*F*_1,28_ = 6.13, $R_{\mathrm{adj}}^{2}$ = .12, *p* < .05; ambient: 0.69 ± 0.11 [mean ± SE] min collecting/min video/flower, warmed: 1.74 ± 0.40 min collecting/min video/flower; see Appendix S1). Despite these results, the amount of pollen deposited by bees on floral stigmas was independent of plant soil moisture (*F*_1,55_ = 0.14, $R_{\mathrm{adj}}^{2}$ = .36, *p* = .71) and temperature (*F*_1,55_ = 0.79, *p* = .38; see Appendix S1). The only factor that affected pollen deposition was the pollination treatment itself: mean pollen deposition was more than twofold higher (*F*_1,55_ = 37.19, *p* < .0001) in hand‐pollinated plants (916 ± 119 total pollen grains per stigma) compared to bee‐pollinated plants (403 ± 25 total pollen grains per stigma; see Appendix S1).

**FIGURE 2:**
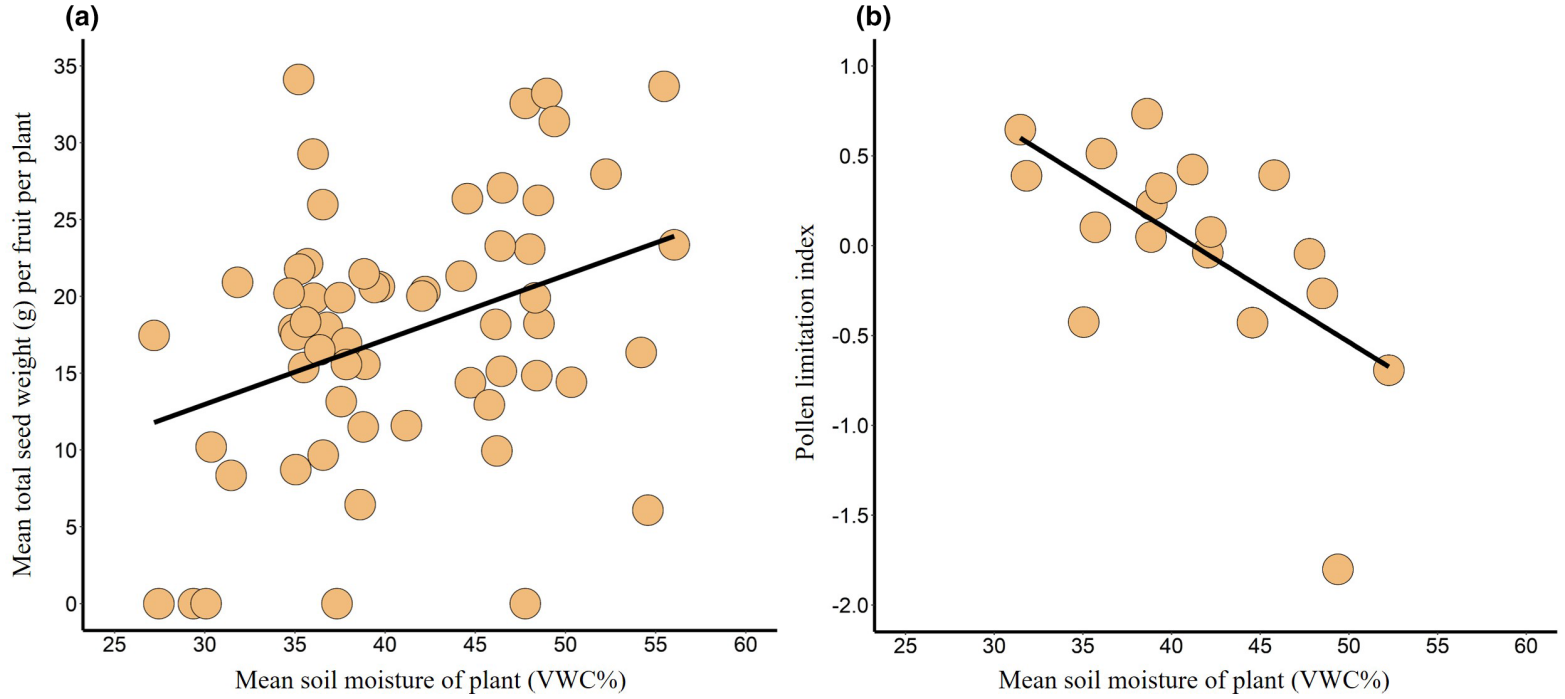
Effects of soil moisture on *Cucurbita pepo* reproductive success in *Experiment I*. (a) Seed set versus plant soil moisture (VWC%, mean percentage volumetric water content) in bee‐pollinated plants. (b) Pollen limitation versus plant soil moisture. Regression lines represent significant relationships; see Results for additional statistical information. The regression in (b) remains significant when the datum in the bottom right portion of the figure is excluded from analysis.

Temperature variation had no effect on either fruit set or seed set (see Appendix S1), but both reproductive variables decreased with decreasing soil moisture (Table 2). Aside from soil moisture, however, no other measured variable, including pollination type (i.e., hand vs. bee), affected fruit set (Table 2; see Appendix S1). In contrast, soil moisture and pollination type had an interactive effect on seed set (Table 2): seed set increased with soil moisture in bee‐pollinated plants (*F*_1,56_ = 8.38, $R_{\mathrm{adj}}^{2}$ = .089, *p* < .01; Figure 2a) but was independent of soil moisture in hand‐pollinated plants (*F*_1,15_ = 2.87, $R_{\mathrm{adj}}^{2}$ = .039, *p* = .11).

**TABLE 2:** The effects of soil moisture, temperature, pollination, and their interactions on fruit set and seed set for *Cucurbita pepo* plants (*n* = 80) in *Experiment I*.

Response Variable	Factor	Estimate	*SE*	*z*	*p*
(a) Fruit set	**Mean soil moisture**	**0.10**	**0.049**	**2.09**	**.037**
	Temperature	1.27	2.49	0.51	.61
	Pollination	−0.12	3.83	−0.033	.97
	Soil moisture × temperature	−0.056	0.062	−0.90	.37
	Soil moisture × pollination	−0.010	0.097	−0.11	.91
	Temperature × pollination	0.37	5.21	0.071	.94
	Soil moisture × temperature × pollination	0.0064	0.13	0.050	.96

Response Variable	Factor	*df*	*F*	*p*	$R_{\mathrm{adj}}^{2}$
(b) Seed set	**Mean soil moisture**	**1,71**	**4.26**	**.043**	**.12**
	Temperature	1,71	0.70	.41	
	**Pollination**	**1,71**	**4.21**	**.044**	
	Soil moisture × temperature	1,71	0.0020	.97	
	**Soil moisture × pollination**	**1,71**	**7.82**	**.0067**	
	Temperature × pollination	1,71	0.25	.62	
	Soil moisture × temperature × Pollination	1,71	0.0020	.97	

*Note*: (a) and (b) present models from separate ANCOVAs. For model (a): *SE*, standard error. For model (b): *df*, degrees of freedom; $R_{\mathrm{adj}}^{2}$, adjusted *R* ^2^ value. Results in bold are statistically significant at *α* = .05.

Pollen limitation decreased with increasing soil moisture (*F*_1,14_ = 10.17, $R_{\mathrm{adj}}^{2}$ = .36, *p* < .01; Figure 2b) but was independent of temperature (*F*_1,14_ = 1.60, *p* = .23; see Appendix S1). There was also no interaction between soil moisture and temperature with respect to pollen limitation (*F*_1,14_ = 0.70, *p* = .42). When we estimated pollen limitation using all bee‐pollinated plants and the mean seed set values of their matched hand‐pollinated plant treatment groups, we found qualitatively similar results as those shown in Figure 2b (see Appendix S1).

### 3.2 Experiment II: Effects of soil‐moisture limitation on pollen viability

**FIGURE 3:**
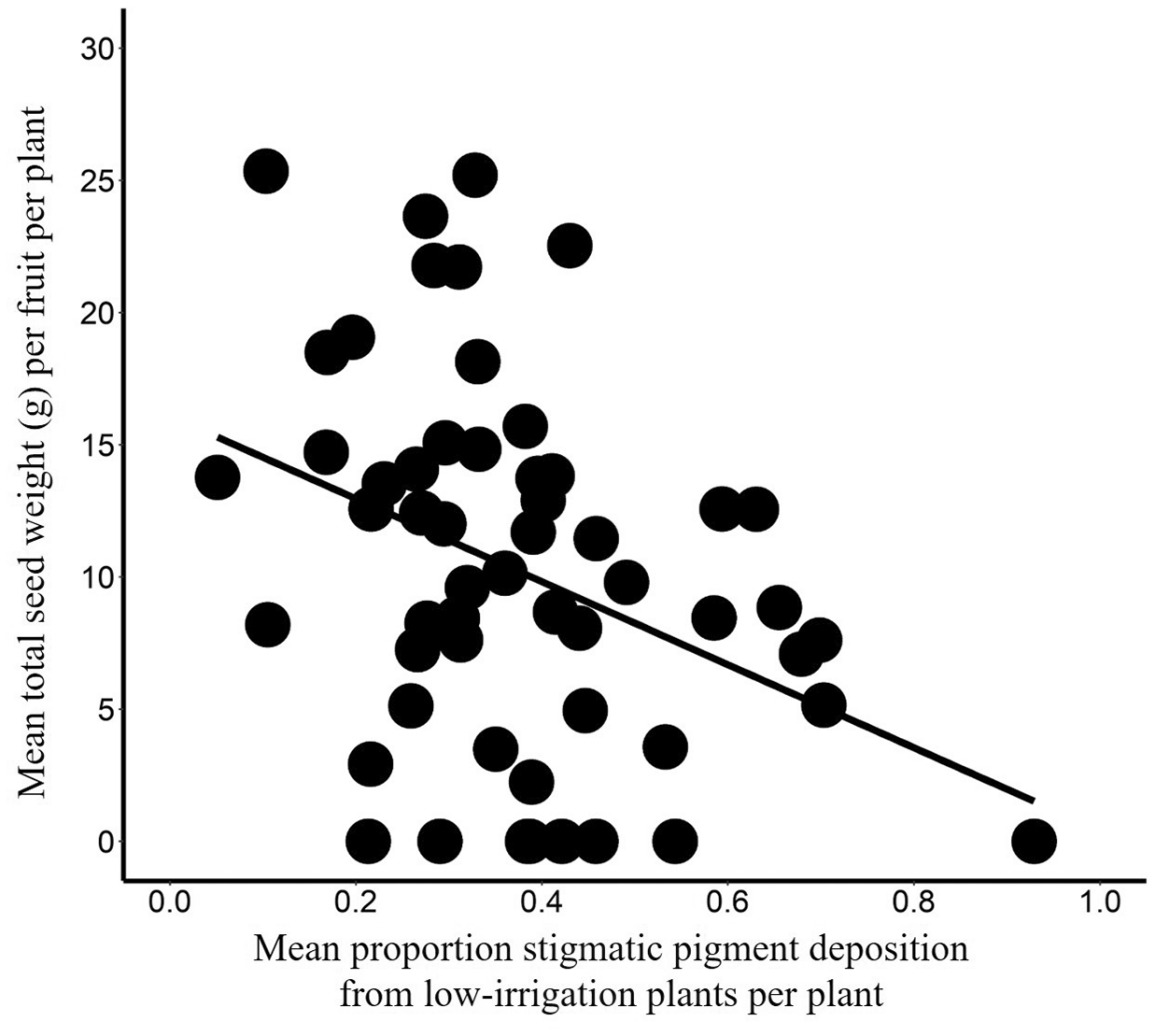
Effect of stigmatic fluorescent pigment deposition from plants grown in the low‐irrigation treatment on seed set in bee‐pollinated *Cucurbita pepo* in *Experiment II*. The regression line represents a significant relationship; see Results for additional statistical information.

This second experiment assessed how pollen source (i.e., irrigation treatment) affected plant reproduction. Seed set decreased with increasing amounts of stigmatic pigment deposition from plants grown in the low‐irrigation treatment (simple linear regression: *F*_1,54_ = 8.82, $R_{\mathrm{adj}}^{2}$ = .12, *p* < .01; Figure 3).

## 4 DISCUSSION

Using experimental manipulations in the field, we tested how warming and soil‐moisture limitation affect the reproduction of bee‐pollinated squash (*Cucurbita pepo*). For floral traits that responded to treatments, effects of low soil moisture were always inhibitory, whereas effects of warmer temperatures included both inhibitory (e.g., smaller corolla size) and stimulatory (e.g., nectar and pollen production) responses. Although honey bee visitation and behavior in flowers responded to treatments in some cases, the amount of pollen deposited by all bees on floral stigmas was independent of treatments. Pollinator behavior (i.e., pollen transfer), however, did indirectly affect squash reproduction as *Experiment II* demonstrated that pollen limitation stemmed at least in part from the transport of pollen that had reduced viability due to soil‐moisture limitation. These results suggest that pollinators mediate the effects of drought by transporting and depositing pollen from plants experiencing soil‐moisture limitation.

In this study, the responses to soil‐moisture limitation were uniformly negative. Observed effects largely mirror those reported in previous studies (Descamps, Quinet, & Jacquemart, 2021), which include reductions in flower size (Burkle & Runyon, 2016; Gallagher & Campbell, 2017; Kuppler & Kotowska, 2021), nectar volume (Gallagher & Campbell, 2017; Kuppler & Kotowska, 2021; Phillips et al., 2018; Waser & Price, 2016), pollen viability (Al‐Ghzawi et al., 2009; Gambel & Holway, 2023), and pollinator visitation (Al‐Ghzawi et al., 2009; Burkle & Runyon, 2016; Gambel & Holway, 2023; Glenny et al., 2018; Rering et al., 2020; Waser & Price, 2016). Yet the impact of soil‐moisture limitation on plant reproductive allocation is not limited to these effects and can also include diminished flower production (Al‐Ghzawi et al., 2009; Burkle & Runyon, 2016; Descamps et al., 2018; Gambel & Holway, 2023; Kuppler & Kotowska, 2021; Phillips et al., 2018), pollen production (Waser & Price, 2016), and nectar sugar content (Waser & Price, 2016), as well as other responses (Descamps, Quinet, & Jacquemart, 2021).

In contrast to the impact of soil‐moisture limitation, the effects of warming were variable. Two of the three significant responses to temperature in *Experiment I* were cases of enhanced performance (e.g., increased nectar volume and pollen production), unlike previous studies that have documented reduced floral resources due to heat stress (Descamps et al., 2018). Hoover et al. (2012), however, also reported that *Cucurbita* increased nectar production in response to experimental warming. Although the degree of warming in the present study was based on current climate projections (Lee et al., 2023; Pachauri et al., 2014), mean summer temperatures at our coastal study site were mild enough (2016–2018 season mean: 25°C) that even experimentally‐warmed conditions did not appear to stress squash plants, which are often grown under much higher temperatures (Loy, 2004; Molinar et al., 1999; Wien et al., 2004). Moreover, the lack of a statistical interaction between the effects of warming and soil‐moisture limitation in *Experiment I* could also be the result of a temperature manipulation that failed to physiologically challenge plants. The effects of these two stressors, however, are known to act independently of one another in other studies focused on plant reproduction (Descamps et al., 2018).

While we expected little change in bee behavior in flowers due to the lack of choice in resource quality in *Experiment I*, honey bees did alter their foraging behaviors in response to the treatments. Compared to specialist squash bees, generalist honey bees collect nectar and pollen from a variety of plant families. In this study, honey bees spent more time collecting pollen from flowers on warmed plants, possibly because warmed plants produced more pollen compared to plants grown at ambient temperatures. Honey bee visitation to female flowers also increased with soil moisture. Other than flower size, however, soil moisture did not affect additional floral traits (at least of those traits measured) that could influence bee behavior, such as nectar volume and concentration. Gambel and Holway (2023) similarly found that honey bee visitation to female flowers increased with increasing soil moisture despite the fact that flower size and nectar production were unaffected by the irrigation treatment. It is possible that honey bees responded to other (unmeasured) floral traits, such as floral volatiles, that may have been affected by plant soil moisture (as in Burkle & Runyon, 2016). More research on how environmental stress affects volatile emissions in squash would help to elucidate this finding.

Here we were able to document pollen limitation resulting from soil‐moisture limitation in a bee‐pollinated plant by restricting pollinator access (e.g., bees could only move pollen among plants in a single experimental group in a given day) and then comparing reproduction between bee‐pollinated and hand‐pollinated plants. Pollen limitation resulting from soil‐moisture limitation is seldom reported in the literature but must be common given that soil‐moisture limitation negatively affects pollen quantity and viability in a variety of plant species (Descamps, Quinet, & Jacquemart, 2021). In the present study, pollen limitation most likely resulted from the transport of lower‐quality pollen from plants experiencing soil‐moisture limitation. In *Experiment II*, for example, squash seed set decreased with increasing amounts of pigment (and, by proxy, pollen) deposited on stigmas by bees from male flowers of plants grown under low soil moisture conditions. Other mechanisms of pollen limitation seem less likely to be as important. Differential pollen production can be ruled out given that the amount of pollen produced by squash was independent of soil moisture. Pollen limitation commonly results from inadequate visitation by pollinators (Goodell et al., 2010; Koski et al., 2018; Moeller et al., 2012), but the amount of pollen that bees deposited on floral stigmas was independent of soil moisture. Like Winsor et al. (1987), however, we did observe a positive relationship between pollen deposition and seed set. Winsor et al. (1987) concluded that more intense pollen competition was the underlying cause of this effect. This mechanism seems to have influenced variation in seed set in our study as well, and thus likely contributed to pollen limitation in the system.

In this study, experimentally‐induced, soil‐moisture limitation led to pollen limitation in cultivated squash plants. Given that drought and warming commonly affect plant reproductive allocation in other plant species (de Manincor et al., 2023; Descamps et al., 2018, 2020; Descamps, Jambrek, et al., 2021; Descamps, Quinet, & Jacquemart, 2021; Koti et al., 2005; Moss & Evans, 2022; Schuchardt et al., 2021; Wu et al., 2022), pollinator‐mediated effects of this kind are likely widespread. Unlike studies that allow pollinators to visit plants grown under a range of physical conditions (Al‐Ghzawi et al., 2009, Burkle & Runyon, 2016, Descamps et al., 2018, Descamps, Jambrek, et al., 2021, Gallagher & Campbell, 2017, Gambel & Holway, 2023, Glenny et al., 2018, Moss & Evans, 2022, Rering et al., 2020, Waser & Price, 2016), the current study presents a climate change scenario in which variation in resource quality may be unavailable to pollinators. Pollinator responses to plants experiencing soil‐moisture limitation could compromise plant reproduction through increased levels of pollen limitation, impacting plant population and community dynamics (Ashman et al., 2004). Experimental studies that consider the joint effects of soil‐moisture limitation and warming on plant reproduction as well as the direct and indirect (i.e., pollinator‐mediated) effects of environmental stress (Höfer et al., 2022; Wu et al., 2022) will add to an understanding of how animal‐pollinated plants across the globe will respond to climate change.

## AUTHOR CONTRIBUTIONS

**Jess Gambel:** Conceptualization (equal); data curation (lead); formal analysis (lead); funding acquisition (lead); investigation (lead); methodology (lead); project administration (lead); supervision (supporting); writing – original draft (lead); writing – review and editing (equal). **David A. Holway:** Conceptualization (equal); formal analysis (supporting); funding acquisition (supporting); methodology (supporting); project administration (supporting); supervision (lead); writing – original draft (supporting); writing – review and editing (equal).

## FUNDING INFORMATION

This research was made possible by funding through the Jeanne Marie Messier Memorial Fund Fellowship, University of California Climate Initiative and Global Food Initiative Fellowships, and the National Institutes of Health CMG Training Grant (No. T32 GM007240).

## CONFLICT OF INTEREST STATEMENT

The authors declare that they have no conflicts of interest.

## Supporting information


Appendix S1.

## Data Availability

Data (Gambel, 2024) are available from figshare: https://doi.org/10.6084/m9.figshare.c.7248592.v1.
