# Supplementary material for: Temperature and soil moisture manipulation yields evidence of drought‐induced pollen limitation in bee‐pollinated squash
Source: Ecol Evol. 2024 Jun 2;14(6):e11400. doi: 10.1002/ece3.11400 (PMC11144714; doi:10.1002/ece3.11400)
Supplement: Supplementary file 1 — Appendix S1. [file ECE3-14-e11400-s001.doc]

**SUPPORTING INFORMATION FOR:**

**Temperature and soil moisture manipulation yields evidence of drought-induced**

**pollen limitation in bee-pollinated squash**

Jess Gambel and David A. Holway

TABLE OF CONTENTS

1. Appendix 1: Methods Page 2
   1. *Experiment I: effects of warming x soil-moisture limitation on*

*bee visitation and plant reproduction* Page 2

- - 1. References Page 5
    2. Tables Page 7
    3. Figures Page 9
  1. *Experiment II: effects of soil-moisture limitation on pollen*

*viability* Page 12

- - 1. Figures Page 13

1. Appendix 2: Results Page 14
   1. *Experiment I: effects of warming x soil-moisture limitation on*

*bee visitation and plant reproduction* Page 14

- - 1. Tables Page 15
    2. Figures Page 26
  1. *Experiment II: effects of soil-moisture limitation on pollen*

*viability* Page 28

- - 1. Figures Page 29

**Appendix 1.** Methods

*Experiment I: effects of warming x soil-moisture limitation on bee visitation and plant reproduction*

***Study system:*** Squash bees and honey bees are the main pollinators of squash at our study site (Gambel & Holway 2023). Solitary squash bees specialize on *Cucurbita* and require pollen from cucurbits to reproduce (Hurd et al. 1971). In our region, squash bees (*Xenoglossa* [formerly *Peponapis* and *Xenoglossa*]; Freitas et al. 2023) visit cultivated squash in gardens and farms as well as native buffalo gourd (*C. foetidissima*). The generalist and eusocial western honey bee (*Apis mellifera*) also visits *Cucurbita* (Hurd et al. 1974, Tepedino 1981, Artz & Nault 2011). Honey bees at our study site are a mix of managed colonies kept at the field station and feral colonies (Kono & Kohn 2015, Hung et al. 2018).

***Experimental setup:*** Squash plants were germinated from seeds planted directly in the ground in June at the University of California, San Diego Biology Field Station, which has a mix of clay and sandy loam soils (USDA NRCS). We added Jobe's Organic Vegetable Fertilizer© (2% nitrogen, 5% phosphorus, 3% potassium; 130 g / plant) to the soil during seeding to aid in plant establishment. After plants matured, we sprayed them with neem oil 2 – 3 times during the season to reduce pest and fungus infestations. We used a drip-line system to irrigate plants every morning. For the first three weeks following the germination of seeds, all plants received 2.0 L water/plant/day to encourage establishment. Plants in the low irrigation group thereafter received 0.67 L water/plant/day. We used a FieldScout TDR 100© soil moisture meter to take 2 – 4 replicate measurements of volumetric water content (VMC%) in the soil 20 cm below each plant one hour after irrigating, 2 – 3 times per week for the duration of the season. Mean volumetric water content varied from 27 - 51% (low irrigation) to 36 - 56% (high irrigation).

***Floral traits:*** Plants flowered from early July to early September. Each afternoon, we prevented bees from visiting a subset of flowers by bagging those flowers ready to open the next morning using Seedburo Treated S27 Shoot Pollinating Bags (5 cm x 2.5 cm x 18 cm; Stoner & Eitzer 2012). On the morning that these flowers opened, we detached them from the plants while still bagged. On these flowers, we measured flower size as the open floral corolla width (Hoover et al. 2012). We used a 2µL pipette to collect nectar from each flower (Stoner & Eitzer 2012). We measured the nectar volume by first centrifuging and then measuring the collected sample using a 200 µL pipette. To measure nectar concentration, we used a 10 µL pipette to remove a 2 µL-sized sample from this collected nectar and placed the sample on a refractometer (Corbet 1978) to measure the BRIX (0-50%) of that sample. We measured up to three samples to obtain a clear reading, which usually occurred with the first sample. We also measured pollen weight for each unvisited male flower. Using a metal spatula, we gently scraped pollen grains off the anthers of the collected male flower (Stoner & Eitzer 2012) into a tared petri dish, which we then weighed using a scale.

***Video recordings of bees:*** We recorded the visitation behaviors of bees within a subset of open flowers for 1.5 h each morning during visitation surveys using Black Box DareDVL Wifi Mini Waterproof Sports Action Dash Cameras. Cameras were placed on tripods approximately 20 – 40 cm away from the flower so as not to obstruct bee visitation. For each female flower video, we estimated the cumulative rates at which bees contacted the stigma per minute video. For each male flower video, we estimated the cumulative rates at which bees contacted the anthers and collected pollen per minute video.

***Hand pollination:*** To hand pollinate individual flowers, we mixed together pollen from two male flowers (from different plants) and then used a cotton swab to generously apply mixed pollen to the entire stigma of the female flower (Winsor et al. 2000). After hand pollination, we re-bagged flowers with breathable mesh bags (Paper Mart standard gold organza [15 cm x 23 cm]) to exclude pollinators while simultaneously permitting flowers to experience conditions like those experienced by flowers open to bees (Kearns & Inouye 1993).

***Pollinated stigmas:*** After collection from plants, pollinated stigmas were stored in the refrigerator in individual vials of 70% ethanol solution.

**REFERENCES**

Artz, D. R., and B. A. Nault. 2011. “Performance of *Apis mellifera*, *Bombus impatiens*, and

*Peponapis pruinosa* (Hymenoptera: Apidae) as Pollinators of Pumpkin.” *Journal of Economic Entomology* 104(4): 1153–61.

Corbet, S. A. 1978. Bee Visits and the Nectar of *Echium vulgare* L. and *Sinapis alba* L.

*Ecological Entomology* 3: 25-37.

Freitas, F., M. Branstetter, V. Franceschini-Santos, A. Dorchin, K. Wright, M.M. López-Uribe,

T. Griswold, F. Silveira, and E. Almeida. 2023. “UCE phylogenomics, biogeography, and

classification of long-horned bees (Hymenoptera: Apidae: Eucerini), with insights on using specimens with extremely degraded DNA.” *Insect Systematics and Diversity* 7(4):1-21.

Gambel, J., and D. A. Holway. 2023. “Divergent Responses of Generalist and Specialist

Pollinators to Experimental Drought: Outcomes for Plant Reproduction.” *Ecology* e4111.

Hoover, S. E., J. J. Ladley, A. A. Shchepetkina, M. Tisch, S. P. Gieseg, and J. M. Tylianakis.

2012. “Warming, CO_2_, and Nitrogen Deposition Interactively Affect a Plant-Pollinator Mutualism.” *Ecology Letters* 15: 227-234.

Hung, K.-L. J., J. M. Kingston, M. Albrecht, D. A. Holway, and J. R. Kohn. 2018. “The

Worldwide Importance of Honey Bees as Pollinators in Natural Habitats.” *Proceedings of the Royal Society B: Biological* *Sciences* 285(1870): 20172140.

Hurd, P. D., E. Gorton Linsley, and A. E. Michelbacher. 1974. “Ecology of the Squash and

Gourd Bee, *Peponapis Pruinosa*, on Cultivated Cucurbits in California (Hymenoptera: Apoidea).” *Smithsonian Contributions to Zoology* 168: 1–17.

Hurd, P. D., E. Gorton Linsley, and T. W. Whitaker. 1971. “Squash and Gourd Bees (*Peponapis,*

*Xenoglossa)* and the Origin of the Cultivated *Cucurbita*.” *Evolution* 25(1): 218–34.

Kearns, C. A., and D. W. Inouye. 1993. *Techniques for Pollination Biologists*. Boulder, CO:

University Press of Colorado.

Kono, Y., and J. R. Kohn. 2015. Range and Frequency of Africanized Honey Bees in California

(USA). *PLoS One* 10(9): e0137407.

Stoner, K. A., and B. D. Eitzer. 2012. “Movement of Soil-Applied Imidacloprid and

Thiamethoxam into Nectar and Pollen of Squash (*Cucurbita pepo*).” *PLoS One* 7(6): e39114.

Tepedino, V. J. 1981. “The Pollination Efficiency of the Squash Bee (*Peponapis pruinosa*) and

the Honey Bee (*Apis mellifera*) on Summer Squash (*Cucurbita pepo*).” *Journal of the Kansas Entomological Society* 54(2): 359–77.

United States Department of Agriculture Natural Resources Conservation Service. Web Soil

Survey. https://websoilsurvey.nrcs.usda.gov/app/. Accessed June 15, 2015.

Winsor, J .A., S. Peretz, and A. G. Stephenson. 2000. Pollen Competition in a Natural Population

of *Cucurbita Foetidissima* (Cucurbitaceae). *American Journal of Botany* 87(4): 527-532.

**Table A1.** The effects of temperature and irrigation manipulations experienced by cultivated *Cucurbita pepo* plants (n = 80) in *Experiment I*.

Response Variable Treatment df *F P* Mean *SE*

Mean average daily **Temperature 1,78 500.80**  **<0.0001**

temperature (˚C) *Warmed* 25.88 0.086

*Ambient* 23.36 0.072

Mean maximum **Temperature 1,78 356.10** **<0.0001**

temperature (˚C) *Warmed* 38.41 0.26

*Ambient* 32.68 0.16

Mean minimum **Temperature 1,78 317.40** **<0.0001**

temperature (˚C) *Warmed* 18.28 0.050

*Ambient* 17.05 0.047

Mean plant soil **Irrigation 1,76 93.92** **<0.0001**

moisture (VWC%) *High* 46.32 0.77

*Low* 36.02 0.72

Temperature 1,76 0.10 0.76

*Warmed* 41.34 1.12

*Ambient* 41.01 1.10

Irrigation x 1,76 0.058 0.81

Temperature

*High x Warmed* 46.61 0.93

*High x Ambient* 46.03 1.24

*Low x Warmed*  36.06 1.15

**Table A1 (continued)**

Response Variable Treatment df *F P* Mean *SE*

*Low x Ambient*  35.99 0.89

Note: Analyses are one-way ANOVAs except for the analysis of mean plant soil moisture for which a two-way ANOVA was performed. df, degrees of freedom; *SE*, standard error of the mean. Results in bold are statistically significant at α = 0.05.

**FIGURE LEGENDS**

**Figure A1.** *Cucurbita pepo* plants grown at the UC San Diego Biology Field Station during June – September 2016 for *Experiment I*. We used a drip irrigation system to water plants and simulated warming using passive, open-top warming chambers. The drip irrigation lines traversed each individual plot, watering the base of each plant. We monitored temperature readings at each individual plant using Thermochron iButtons© housed in PVC piping. Individual plants were randomly assigned to one of four treatment groups: ambient temperature, high irrigation; warm temperature, high irrigation; ambient temperature, low irrigation; or warm temperature, low irrigation. Within each treatment group, plants were then either open-pollinated by bees (n = 15) or strictly pollinated by hand (n = 5).

**Figure A2.** Daily flower production from June 29 – September 22 for *Cucurbita pepo* plants grown in 2016 for *Experiment I*. Flower production includes total flowers produced from all treatment groups per day of the experiment.

**Figure A1**


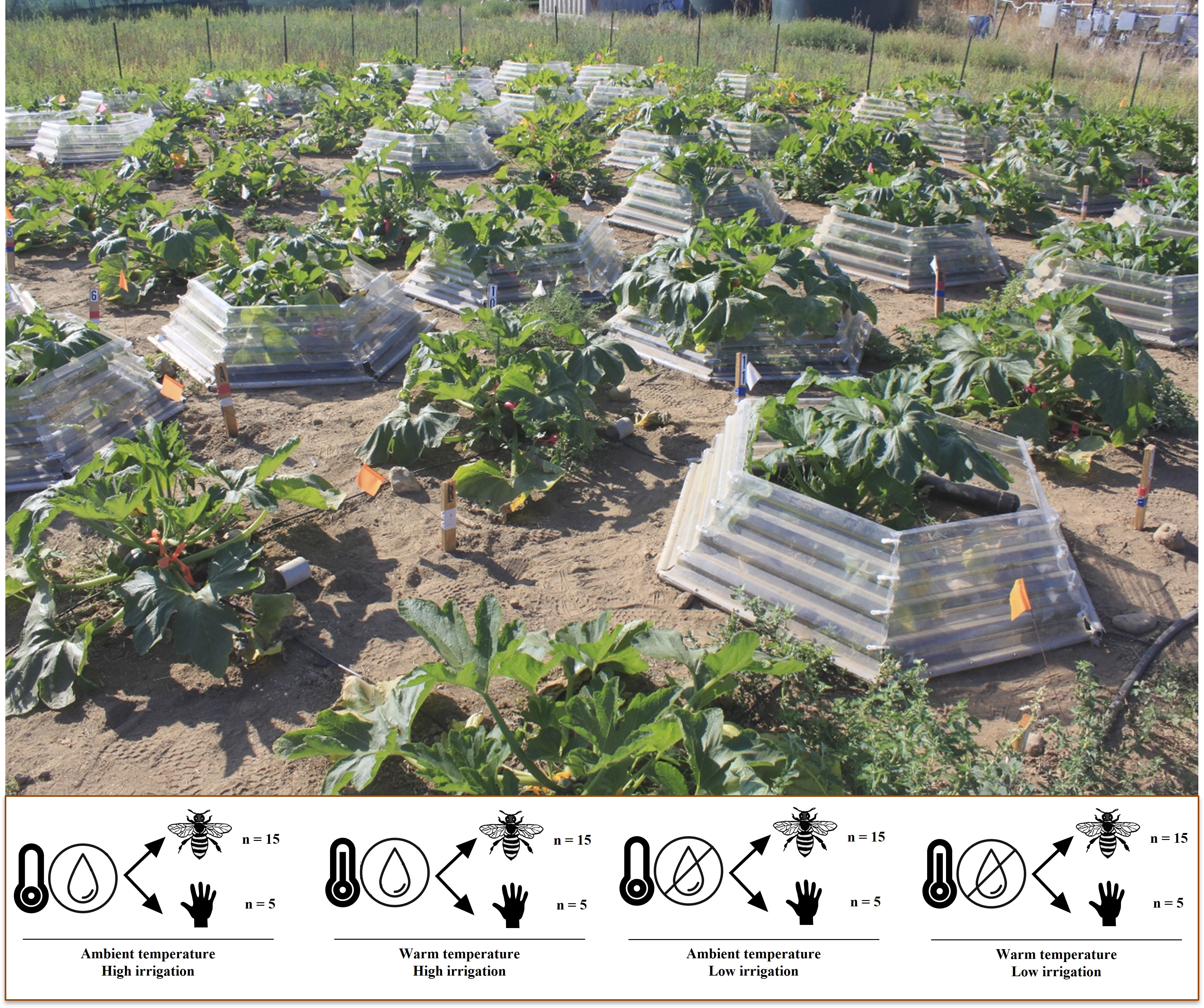


**Figure A2**

**
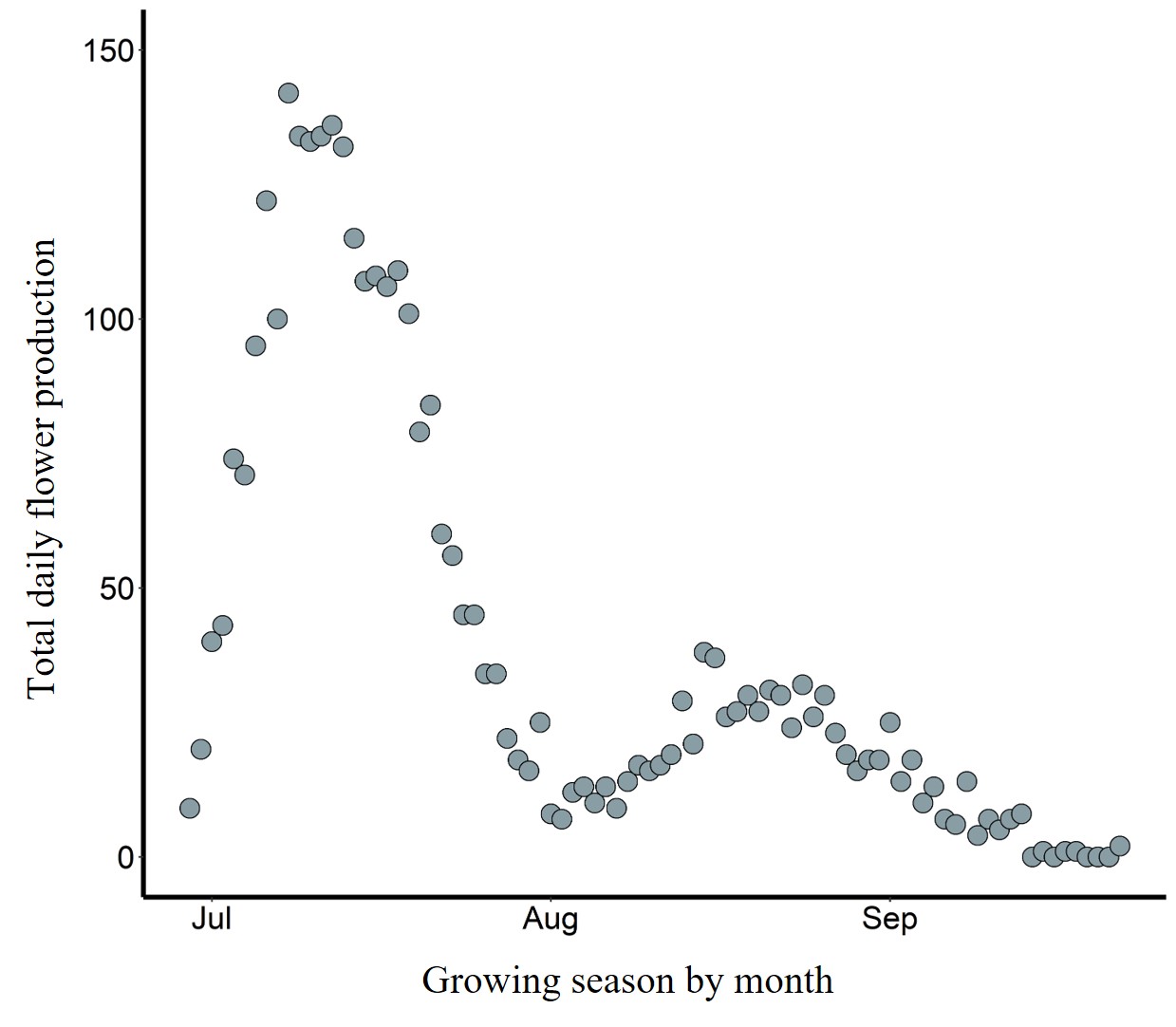
**

*Experiment II: effects of soil-moisture limitation on pollen viability*

***Pollinated stigmas:*** Before allowing bees access to flowers each morning, we applied powdered DayGlo© fluorescent pigments (colors: aurora pink and horizon blue) to open male flowers. On a given day, male flowers on plants in each irrigation group received different pigment colors; color assignments were switched daily to control for any color preferences exhibited by bees. Collected stigmas were stored in 100% ethanol solution instead of 70% solution (as in *Experiment I*) due to issues with stigmas becoming contaminated with mold in the 70% solution. Additionally, we counted all pollen grains deposited on stigmas (instead of averaging the count from 2 lobes per stigma, as in *Experiment I*) to be consistent with counting entire stigmas for pigment particles.

**FIGURE LEGENDS**

**Figure A3.** Daily flower production from July 16 – September 8 for *Cucurbita pepo* plants grown in 2018 for *Experiment II*. Flower production includes total flowers produced from all treatment groups per day of the experiment.

**
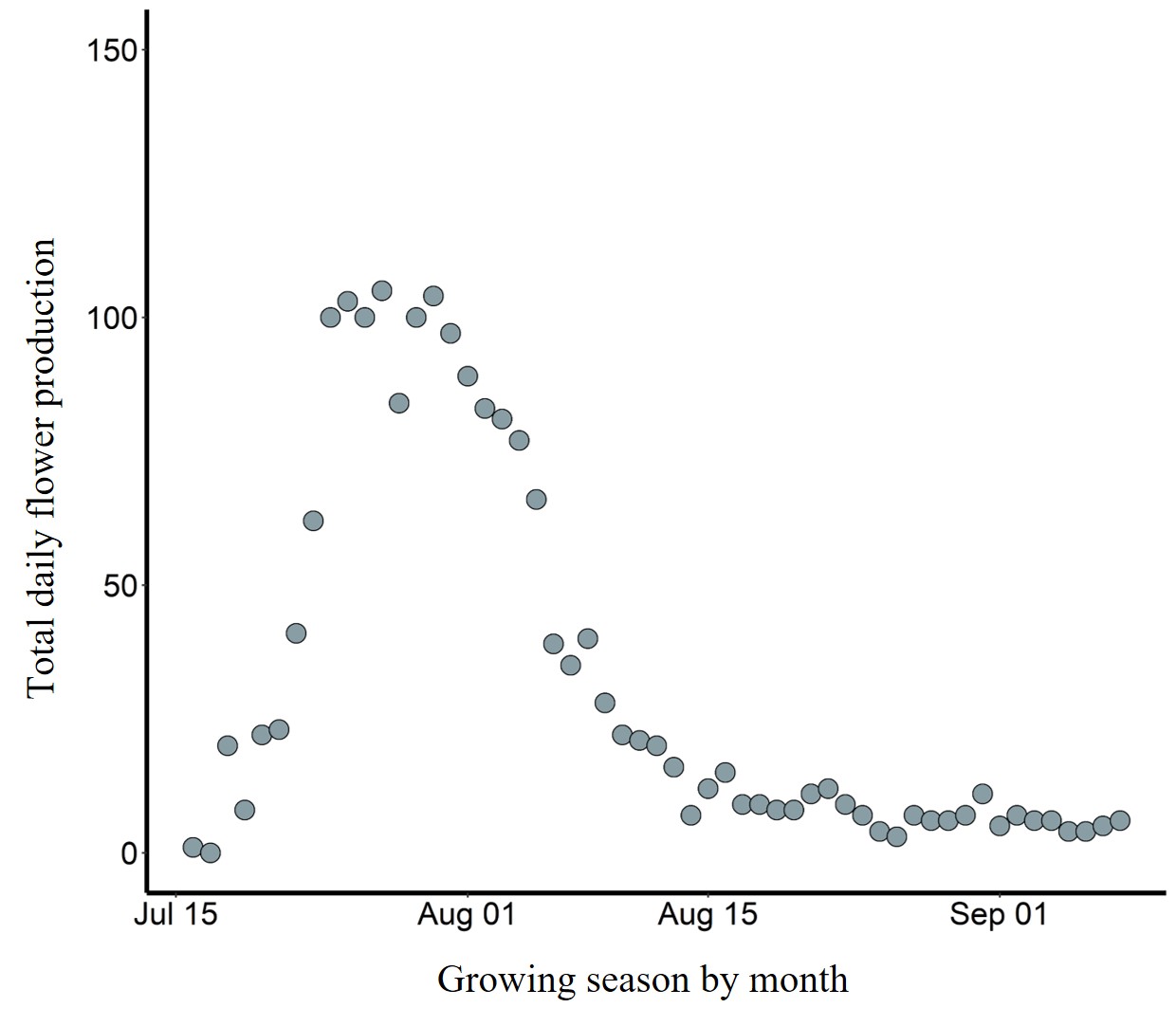
**

**Appendix 2.** Results

*Experiment I: effects of warming x soil-moisture limitation on bee visitation and plant reproduction*

**Table A2.** Means and standard errors for floral traits in bee-pollinated *Cucurbita pepo* (n = 60 plants) in warmed and ambient temperature treatments in *Experiment I*.

Response Variable Temperature Treatment Mean Standard Error

Total male flowers Warmed 22.37 1.73

Ambient 19.07 1.49

Total female flowers Warmed 11.27 1.28

Ambient 11.27 1.11

Male flower size **Warmed 51.97 1.22**

(corolla width in mm) **Ambient 56.14 1.56**

Female flower size **Warmed 56.49 1.70**

(corolla width in mm) **Ambient 63.34 1.68**

Nectar volume (µL) **Warmed 39.08 2.86**

in male flowers **Ambient 32.55 1.96**

Nectar volume (µL) Warmed 59.74 4.35

in female flowers Ambient 70.16 3.97

Nectar concentration Warmed 31.08 0.55

(BRIX) in male flowers Ambient 31.62 0.63

Nectar concentration Warmed 33.94 0.69

(BRIX) in female flowers Ambient 35.48 0.49

Pollen weight (mg) **Warmed 18.45 1.05**

in male flowers **Ambient 15.36 0.83**

Note: All plant measurements (except flower count) were averaged across the flowering season. Results in bold are statistically significant at α = 0.05.

**Table A3.** The effects of soil moisture, temperature, and their interaction on bee visitation and bee behavior in *Cucurbita pepo* flowers (male: n = 33 plants; female: n = 27 plants) in *Experiment I*.

Response Variable Treatment df *F P R^2^*_adj_

(A) Male flowers

*Apis mellifera* (honey bee)

Visits per minute Mean soil moisture 1,28 0.69 0.41 0.00

per flower Temperature 1,28 0.18 0.68

Soil moisture x temperature 1,28 0.00 1.00

Cumulative anther Mean soil moisture 1,28 0.21 0.65 0.033

contact per minute Temperature 1,28 0.56 0.46

per flower* Soil moisture x temperature 1,28 3.30 0.080

Cumulative pollen Mean soil moisture 1,28 1.22 0.28 0.12

collecting per minute **Temperature 1,28 6.13 0.020**

per flower* Soil moisture x temperature 1,28 0.0030 0.96

*Xenoglossa* spp. (squash bee)

Visits per minute Mean soil moisture 1,28 0.30 0.59 0.028

per flower^+^ Temperature 1,28 1.04 0.32

Soil moisture x temperature 1,28 2.57 0.12

Cumulative anther Mean soil moisture 1,28 0.017 0.90 0.00

contact per minute Temperature 1,28 0.0070 0.93

per flower* Soil moisture x temperature 1,28 2.00 0.17

**Table A3 (continued)**

Response Variable Treatment df *F P R^2^*_adj_

Cumulative pollen Mean soil moisture 1,28 0.47 0.50 0.00 collecting per minute Temperature 1,28 0.039 0.85

per flower* Soil moisture x temperature 1,28 0.50 0.48

(B) Female flowers

*Apis mellifera* (honey bee)

Visits per minute **Mean soil moisture 1,23 4.79 0.039 0.13**

per flower Temperature 1,23 0.061 0.81

Soil moisture x temperature 1,23 1.96 0.18

Cumulative stigma Mean soil moisture 1,23 1.11 0.30 0.0068

contact per minute Temperature 1,23 1.06 0.31

per flower* Soil moisture x temperature 1,23 1.01 0.33

*Xenoglossa* spp. (squash bee)

Visits per minute Mean soil moisture 1,23 1.23 0.28 0.021

per flower^+^ Temperature 1,23 0.19 0.66

Soil moisture x temperature 1,23 2.13 0.16

Cumulative stigma Mean soil moisture 1,23 0.41 0.53 0.00

contact per minute Temperature 1,23 0.068 0.80

per flower* Soil moisture x temperature 1,23 1.07 0.31

Note: Models are ANCOVAs. To improve normality of the residuals, response variables that

**Table A3 (continued)**

were right-skewed were either log_10_ (*) or arcsine square-root (^+^) transformed. df, degrees of freedom; *R*^2^_adj_ , adjusted *R*^2^ value. Results in bold are statistically significant at α = 0.05.

**Table A4.** Means and standard errors for bee visitation and bee behavior in *Cucurbita pepo* flowers (male: n = 33 plants; female: n = 27 plants) in warmed and ambient temperature treatments in *Experiment I*.

Response Variable Temperature Treatment Mean Standard Error

(A) Male flowers

*Apis mellifera* (honey bee)

Visits per minute Warmed 0.34 0.028

per flower Ambient 0.37 0.044

Cumulative anther Warmed 5.25 2.02

contact per minute Ambient 3.79 1.28

per flower

Cumulative pollen **Warmed 1.74 0.40**

collecting per minute **Ambient 0.69 0.11**

per flower

*Xenoglossa* spp. (squash bee)

Visits per minute Warmed 0.085 0.017

per flower Ambient 0.14 0.041

Cumulative anther Warmed 4.53 2.04

contact per minute Ambient 4.80 2.54

per flower

Cumulative pollen Warmed 0.21 0.11

collecting per minute Ambient 0.22 0.091

per flower

**Table A4 (continued)**

Response Variable Temperature Treatment Mean Standard Error

(B) Female flowers

*Apis mellifera* (honey bee)

Visits per minute Warmed 0.42 0.040

per flower Ambient 0.37 0.061

Cumulative stigma Warmed 3.35 0.49

contact per minute Ambient 5.27 1.56

per flower

*Xenoglossa* spp. (squash bee)

Visits per minute Warmed 0.12 0.035

per flower Ambient 0.12 0.043

Cumulative stigma Warmed 7.92 3.54

contact per minute Ambient 4.40 1.41

per flower

Note: Results in bold are statistically significant at α = 0.05.

**Table A5.** The effects of soil moisture, temperature, pollination, and their interactions on stigmatic pollen deposition in *Cucurbita pepo* plants (n = 63) in *Experiment I*.

Response Variable Factor df *F P R^2^*_adj_ Mean *SE*

Pollen Mean soil moisture 1,55 0.14 0.71 0.36

deposition Temperature 1,55 0.79 0.38

(pollen grains *Warmed* 545.41 69.52

per stigma) *Ambient* 588.87 75.51

**Pollination 1,55 37.19 <0.0001**

*Bee* 403.35 24.52

*Hand* 916.03 119.43

Soil moisture x 1,55 0.48 0.49

temperature

Soil moisture x 1,55 2.47 0.12

pollination

Temperature x 1,55 0.49 0.49

pollination

*Warmed x Bee* 375.74 32.13

*Warmed x Hand* 935.65 163.17

*Ambient x Bee* 435.10 37.19

*Ambient x Hand* 896.40 183.08

Soil moisture x 1,55 0.45 0.51

temperature x

pollination

**Table A5 (continued)**

Note: Models are ANCOVAs. Pollen deposition values were right-skewed and thus log_10_ transformed to improve normality of the residuals. df, degrees of freedom; *R*^2^_adj_ , adjusted *R*^2^ value; *SE*, standard error of the mean. Results in bold are statistically significant at α = 0.05.

**Table A6.** Means and standard errors for fruit set and seed set in *Cucurbita pepo* plants (n = 80) in *Experiment I*.

Response Variable Treatment Mean Standard Error

(A) Fruit set Temperature

Surviving fruits *Warmed* 1.28 0.12

(per plant) *Ambient* 1.30 0.11

Pollination

*Bee* 1.27 0.098

*Hand* 1.35 0.13

Temperature x pollination

*Warmed x Bee* 1.20 0.14

*Warmed x Hand* 1.50 0.22

*Ambient x Bee* 1.33 0.14

*Ambient x Hand* 1.20 0.13

Aborted fruits Temperature

(per plant) *Warmed* 1.58 0.21

*Ambient* 0.73 0.13

Pollination

*Bee* 1.08 0.16

*Hand* 1.35 0.23

**Table A6 (continued)**

Response Variable Treatment Mean Standard Error

Temperature x pollination

*Warmed x Bee* 1.57 0.26

*Warmed x Hand* 1.60 0.37

*Ambient x Bee* 0.60 0.15

*Ambient x Hand* 1.10 0.28

(B) Seed set Temperature

(grams per fruit *Warmed* 19.49 1.41

per plant) *Ambient* 17.92 1.33

**Pollination**

***Bee* 17.59 1.10**

***Hand* 22.26 1.87**

Temperature x pollination

*Warmed x Bee* 18.18 1.65

*Warmed x Hand* 23.43 2.44

*Ambient x Bee* 17.01 1.47

*Ambient x Hand* 20.96 2.96

Note: Results in bold are statistically significant at α = 0.05.

**Table A7.** The effects of soil moisture, temperature, and their interaction on pollen limitation in *Cucurbita pepo* in *Experiment I*.

Response Variable Treatment df *F P R^2^*_adj_ Mean *SE*

(A) Pollen Limitation **Mean soil moisture 1,14 10.17 <0.01 0.36**

(as summarized in Temperature 1,14 1.60 0.23

the Results) *Warmed* 0.10 0.12

*Ambient* -0.080 0.26

Soil moisture x 1,14 0.70 0.42

temperature

(B) Pollen Limitation **Mean soil moisture 1,51 12.32 <0.001 0.15**

(using estimates for Temperature 1,51 0.0030 0.95

all bee-pollinated *Warmed* 0.13 0.062

plants, n = 55) *Ambient* 0.12 0.067

Soil moisture x 1,51 0.28 0.60

temperature

Note: See Methods for additional details on how the analysis in (A) is based on a slightly different method of estimating pollen limitation compared to that used in (B). df, degrees of freedom; *R*^2^_adj_, adjusted *R*^2^ value; *SE*, standard error of the mean. Results in bold are statistically significant at α = 0.05.

**FIGURE LEGENDS**

**Figure A4.** Effects of temperature and soil moisture on honey bee (*Apis mellifera*) behavior in *Cucurbita pepo* plants from *Experiment I*. See Results and Appendix 2: Table A3 for additional statistical information. (a) Visitation by *Apis* to female flowers *versus* plant soil moisture (VWC%, mean percentage volumetric water content). Each data point represents a separate plant for which we video-recorded a female flower. Regression line shown is statistically significant. (b) Pollen collection by *Apis* in male flowers *versus* experimental temperature treatment. Asterisk (*) shown represents statistical significance between treatment groups. Box plots include the interquartile range with median line and whiskers that show quartiles ± 1.5 x interquartile range; point represents an extreme value.

**Figure A4**


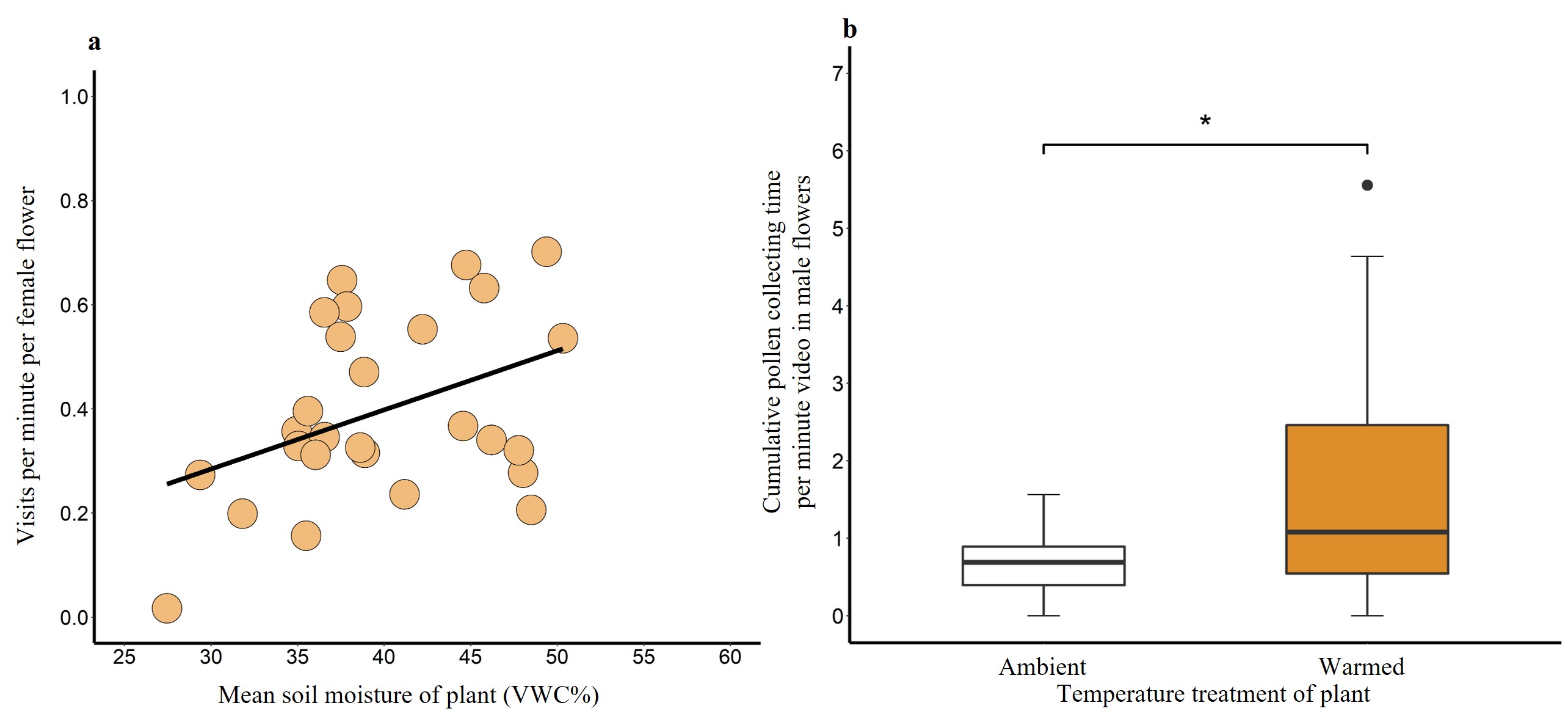


*Experiment II: effects of soil-moisture limitation on pollen viability*

***Seed set:*** Seed set decreased with decreasing stigmatic pollen deposition (simple linear regression: F_1,46_ = 9.88, R^2^_adj_ = 0.16, P < 0.01; Fig. A5a). Additionally, as in Experiment I, seed set decreased with decreasing soil moisture of the recipient plant (simple linear regression: F_1,54_ = 57.92, R^2^_adj_ = 0.51, P < 0.0001; Fig. A5b).

**FIGURE LEGEND**

**Figure A5.** Effects of pollen deposition and soil moisture on seed set in bee-pollinated *Cucurbita pepo* plants from *Experiment II*. Seed set *versus* (a) stigmatic pollen deposition, and (b) plant soil moisture (VWC%, mean percentage volumetric water content of the recipient plant). Regression lines represent significant relationships; see Appendix 2 for additional statistical information.

**Figure A5**

**
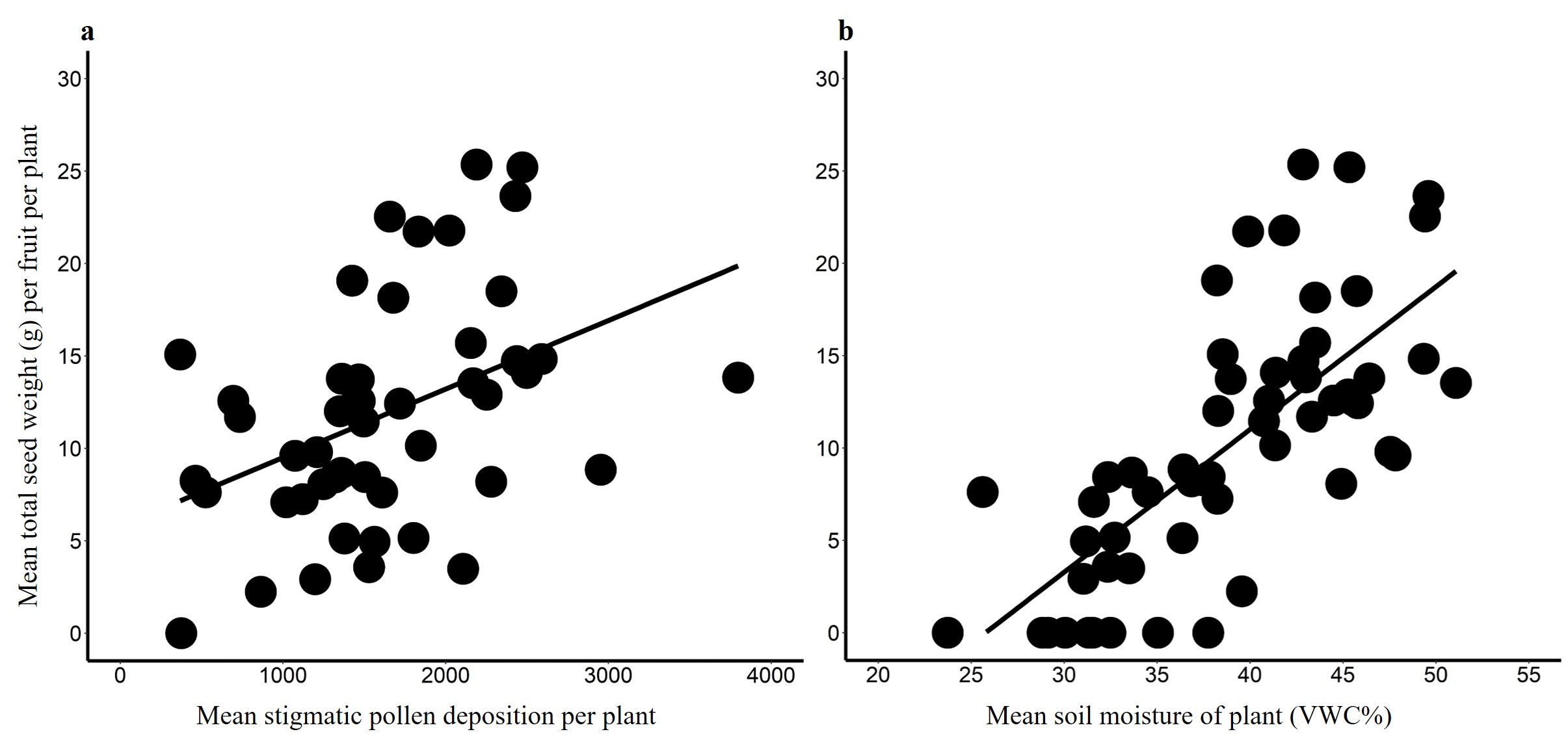
**
